# Metagenomic clustering links specific metabolic functions to globally relevant ecosystems

**DOI:** 10.1128/msystems.00573-24

**Published:** 2024-07-09

**Authors:** Zachary Flinkstrom, Samuel Bryson, Pieter Candry, Mari-Karoliina H. Winkler

**Affiliations:** 1 Department of Civil and Environmental Engineering, University of Washington, Seattle, Washington, USA; 2 Phase Genomics, Seattle, Washington, USA; 3 Laboratory of Systems & Synthetic Biology, Wageningen University & Research, Wageningen, Netherlands; California State University, Northridge, Northridge, California, USA

**Keywords:** metagenomics, microbial ecology, environmental microbiology, functional genes, GC content, metagenome clustering, biogeochemistry

## Abstract

Metagenomic sequencing has advanced our understanding of biogeochemical processes by providing an unprecedented view into the microbial composition of different ecosystems. While the amount of metagenomic data has grown rapidly, simple-to-use methods to analyze and compare across studies have lagged behind. Thus, tools expressing the metabolic traits of a community are needed to broaden the utility of existing data. Gene abundance profiles are a relatively low-dimensional embedding of a metagenome’s functional potential and are, thus, tractable for comparison across many samples. Here, we compare the abundance of KEGG Ortholog Groups (KOs) from 6,539 metagenomes from the Joint Genome Institute’s Integrated Microbial Genomes and Metagenomes (JGI IMG/M) database. We find that samples cluster into terrestrial, aquatic, and anaerobic ecosystems with marker KOs reflecting adaptations to these environments. For instance, functional clusters were differentiated by the metabolism of antibiotics, photosynthesis, methanogenesis, and surprisingly GC content. Using this functional gene approach, we reveal the broad-scale patterns shaping microbial communities and demonstrate the utility of ortholog abundance profiles for representing a rapidly expanding body of metagenomic data.

**IMPORTANCE:**

Metagenomics, or the sequencing of DNA from complex microbiomes, provides a view into the microbial composition of different environments. Metagenome databases were created to compile sequencing data across studies, but it remains challenging to compare and gain insight from these large data sets. Consequently, there is a need to develop accessible approaches to extract knowledge across metagenomes. The abundance of different orthologs (i.e., genes that perform a similar function across species) provides a simplified representation of a metagenome’s metabolic potential that can easily be compared with others. In this study, we cluster the ortholog abundance profiles of thousands of metagenomes from diverse environments and uncover the traits that distinguish them. This work provides a simple to use framework for functional comparison and advances our understanding of how the environment shapes microbial communities.

## INTRODUCTION

Environmental metagenomics has uncovered the diversity of microbial life driving nutrient cycling in different ecosystems. An overarching goal of the field has been to connect environmental conditions to genomic characteristics to identify “who’s there?” and “what are they doing?”. As DNA sequencing technology has become more accessible, the amount of metagenomic data has ballooned. For example, the Joint Genome Institute’s Integrated Microbial Genomes and Metagenomes (JGI IMG/M) database contains more than 30,000 metagenomes (1) and the European Nucleotide Archive (ENA) contains upwards of 50,000 metagenomes (2). Large cross-study analyses of this data can increase our understanding of the patterns in microbiome function across environments. However, these efforts are resource intensive given the size and complexity of the data. To illustrate this, consider the hierarchical nature of a processed metagenome consisting of sequencing reads assembled into contigs which contain genes that are assigned a function, taxonomy, and relative abundance. Furthermore, contigs can be binned into metagenome-assembled genomes (MAGs) based on characteristics such as tetranucleotide frequency and read coverage (3, 4).

Genome-centric analysis of metagenomes can shed light on the metabolic potential of uncultivated microorganisms. For instance, MAGs have expanded our view of nitrogen fixation in the surface ocean (5), methane metabolism in archaea (6–8), and carbon degradation in permafrost (9). Multiple large collections of MAGs have been compiled using metagenomes from the human gut (10), anaerobic digestors (11), and a mix of different environments (12). While these collections provide a valuable resource for the exploration of the vast functional and taxonomic diversity of microbiomes, they do not reveal the functional composition of the whole community or enable facile comparison across samples. To this end, a gene-centric approach may be preferred.

Early metagenomic studies, using limited sample sets, observed how ortholog abundance differed across environments according to the geochemical context (13, 14). Studies of specific ecosystems have linked functional variation to parameters such as temperature, pH, and latitude within topsoil (15), permafrost soil (16), and ocean microbiomes (17, 18). Together, these studies demonstrate how ortholog abundance is a valuable metric for representing a metagenome and linking it to its source environment. In addition, abundance profiles have a simple data structure that allows for large-scale comparisons and the placement of new data in the context of existing samples.

In this study, we evaluate the characteristics of KEGG Ortholog Group (KO) abundance profiles across a large and diverse set of metagenomes from JGI IMG/M. We use unsupervised clustering to show how functional potential stratifies samples by environment and identify the marker KOs that differentiate sample groupings. Interestingly, we note differences in GC content between functional clusters and explore the KOs associated with this trait. The compiled data set can be explored and expanded upon with new metagenomes. Overall, this study provides insight into how environment shapes the functional composition of microbial communities and supports the use of ortholog abundance profiles for understanding metagenomic data.

## MATERIALS AND METHODS

### Data retrieval and preprocessing

Ortholog abundance data were retrieved from the JGI IMG/M database in January 2022. Metagenomes were selected if they had (i) no data use restrictions, (ii) were sequenced and processed through JGI standard workflows (19), (iii) contained more than 100,000 genes assembled and more than 30% of the genes had a KEGG KO (Kyoto Encyclopedia of Genes and Genomes Orthology) (20) annotation assigned, (iv) had estimated gene copy data, and (v) were not a combined assembly of multiple read libraries. The JGI IMG/M functional profile tool was then used to retrieve the estimated gene copies, based on read mapping coverage, of all KOs for each sample. In other words, the KO abundance count represents the sum of the abundance of every instance of that KO in a metagenome. All genes without a KO assignment were excluded from the downstream analyses.

Additionally, the metadata associated with each metagenome was exported. The sample ecosystem label was defined by the “Ecosystem Type” metadata field if more than 100 samples shared the label, otherwise the broader “Ecosystem” field was used to describe the sample. This was done to reduce the number of ecosystem categories containing few samples. JGI taxonomic composition report tables describing contig-level taxonomic assignment were available for 4,988 of the 6,539 total metagenomes. These data provided a count of contigs assigned to different domains and phyla in each metagenome. Taxonomic assignment was determined based on the lineage that received a majority of hits in a database homology search of the contig’s protein-coding genes (19).

Subsequent data processing and analysis was performed in Python Jupyter Notebooks that are available in the study’s github repository (https://github.com/zflink/Metagenome_functional_clustering). The SCANPY (21) gene expression data analysis Python package and its ANNDATA data structure class were used to facilitate processing and exploration of the KO profiles and associated metadata. While SCANPY was developed for the analysis of single-cell RNA sequencing data, its data structure, analysis, and plotting tools remain useful for any high-dimensional data set that has lots of metadata associated with its constituent variables and observations. To construct the ANNDATA object, ortholog abundance tables, sample metadata, and KO names were first loaded into pandas dataframes before being passed to the “anndata.AnnData” constructor function with ortholog abundance as the data matrix, sample metadata as the observations table, and KO names as the variable table. Rare or nonexistent KOs were removed from the data set if they were present in less than 10% of samples. The resulting KO abundance profiles were center-log_2_ ratio (clr) normalized on a per sample basis based on the method from ALDEx2 (22), yielding normalized abundance values per KO in each sample.

### Unsupervised clustering

We explored the unsupervised clustering of the data set with hierarchical clustering using the Ward variance minimization algorithm, implemented in scipy (23), and k-means clustering, implemented in sci-kit learn (24). To evaluate the performance of the two methods and to find the optimal number of clusters, we used a bootstrapping approach where the data set was randomly sampled and clustered 100 times. Bootstrap cluster performance was evaluated using the average silhouette score and adjusted rand index. To prevent overfitting, the final k-means model used for clustering was trained using a random sample of the data set containing half of the samples. For the sub-clustering analysis, the data were subset based on cluster assignment, and then k-means clustering was performed again to find two sub-clusters within each of the three main clusters. Clusters were visualized on the uniform manifold approximation and projection (UMAP) (25) of the normalized data set implemented in SCANPY (21) using 50 neighbors and 5 principal components. UMAP is a nonlinear dimensionality reduction technique that creates a less overcrowded projection of the data set compared to a linear method like principal component analysis (PCA) (25).

### Marker KO analysis

Significant differences in normalized KO abundance across clusters were defined using an ANOVA-like Kruskal-Wallis *H* test [implemented in scipy (23)] followed by Dunn’s test for post-hoc multiple comparisons [implemented in scikit-posthocs (26)]. Cluster-specific marker genes were defined if a KO had a Bonferroni-corrected *P*-value < 0.001 for all Dunn’s tests and had a median log_2_(fold-change) of greater than 2 compared to every other cluster. The false discovery rate of the *P*-value and fold-change thresholds was estimated using permutation testing, which entailed randomly assigning cluster membership 100 times followed by calculating Dunn’s test *P*-values and median fold-changes for each KO which produced no false positive marker KOs. Marker KOs were mapped to their associated pathways by searching for their ortholog number in the KEGG pathway definition file downloaded from the KEGG database.

### GC content KO correlation

Differences in assembled metagenome GC content (derived from JGI data set metadata) across clusters were evaluated like the marker KOs with an ANOVA-like Kruskal-Wallis *H* test followed by Dunn’s test for pairwise comparisons between clusters. Correlated KOs were evaluated by fitting a linear regression for each set of KO abundances against GC content. The Pearson correlation coefficient was used as a measure of goodness of fit, and significance was evaluated using the Wald test as implemented in Scipy’s linregress function.

## RESULTS

### Ortholog abundance profiles cluster metagenomes by ecosystem

**Fig 1:**
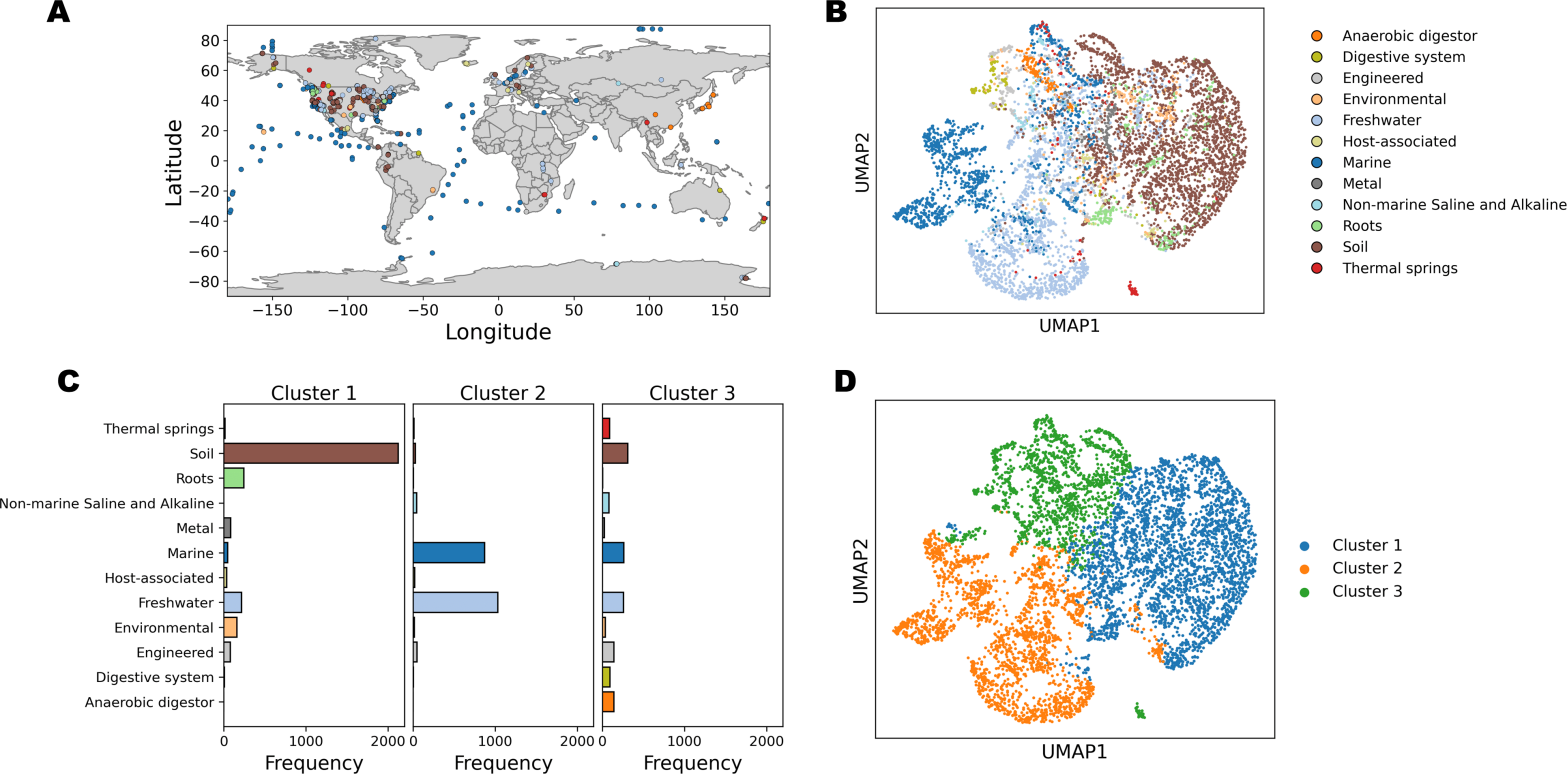
Global distribution of data set and functional clustering. (**A**) Sampling location colored by ecosystem label plotted on a global map. (**B**) UMAP projection of the normalized KO profiles colored by ecosystem label. (**C**) Frequency of different ecosystem labels in the three k-means clusters. (**D**) The same UMAP projection as panel (**B**) but colored by cluster membership.

The final data set consisted of 6,539 metagenomes with 9,871 KO gene orthologs, representing a total of 4.85 terabases of assembled sequence data with an average of 742 ± 10.8 megabases per sample (Fig. S1A). The metagenomes contained an average of 1.50 million genes with 38.8% having an associated KO annotation (Fig. S1B and C). The data set contained samples from across the globe although they predominantly originated from the United States (Fig. 1A). A variety of ecosystems are represented with soils (*n* = 2,464), freshwater (*n* = 1,506), and marine (*n* = 1,184) being the most prevalent.

The UMAP of normalized KO abundance, which aids in the visualization of this high dimensional data set, showed separation of aquatic and terrestrial ecosystems with some overlap occurring in the center (Fig. 1B). On the aquatic side, there was a distinct grouping of freshwater and marine samples. To investigate the groupings of metagenomes in this functional gene space, independent of the ecosystem label, we performed unsupervised clustering on the normalized KO abundance profiles, testing both k-means and hierarchical clustering using Ward’s method for their abilities to produce consistent and cohesive clusters as measured by the adjusted Rand Index and Silhouette Coefficient. Bootstrapping of the data set over a range of cluster numbers showed that k-means performed consistently better than Ward’s hierarchical clustering in terms of consistency and cohesiveness and showed the optimum number of clusters was three (Fig. S2). Accordingly, we proceeded with analyzing three clusters generated by the k-means method.

Membership of the resulting clusters followed environmental origin with some exceptions. For example, Cluster 1 was dominated by samples from soil environments (70.7%) though it also contained samples associated with plant roots (8.1%) and some freshwater samples (7.1%). Cluster 2 consisted almost entirely of freshwater (49.7%) and marine (42.0%) samples. Lastly, Cluster 3 contained a mixture of ecosystem categories. It is worth noting that all 142 samples associated with anaerobic digestors and 93 out of 106 samples associated with digestive systems were found in Cluster 3 (Fig. 1C). Plotting cluster membership on the UMAP projection illustrated how the high-dimensional functional gene space is split by the k-means algorithm and confirms the broad grouping of samples based on environmental origin (Fig. 1D). Still, based on the Silhouette Coefficient, the k-means approach produced more cohesive groups of samples compared to using the ecosystem label (Fig. S3).

The characteristics of the underlying metagenomes within each cluster were compared to evaluate any differences in sequence assembly and annotation. Cluster 1 metagenomes had a lower proportion of sequencing reads assembled and lower average coverage compared to Cluster 2 and 3 (Fig. S4A and C), which is not surprising given the exceptional diversity of soil microbial communities (15). Total gene count and the proportion of genes with an assigned KO were more consistent across the clusters (Fig. S4B and D).

### Marker KO analysis reveals metabolic functions that differentiate clusters

**Fig 2:**
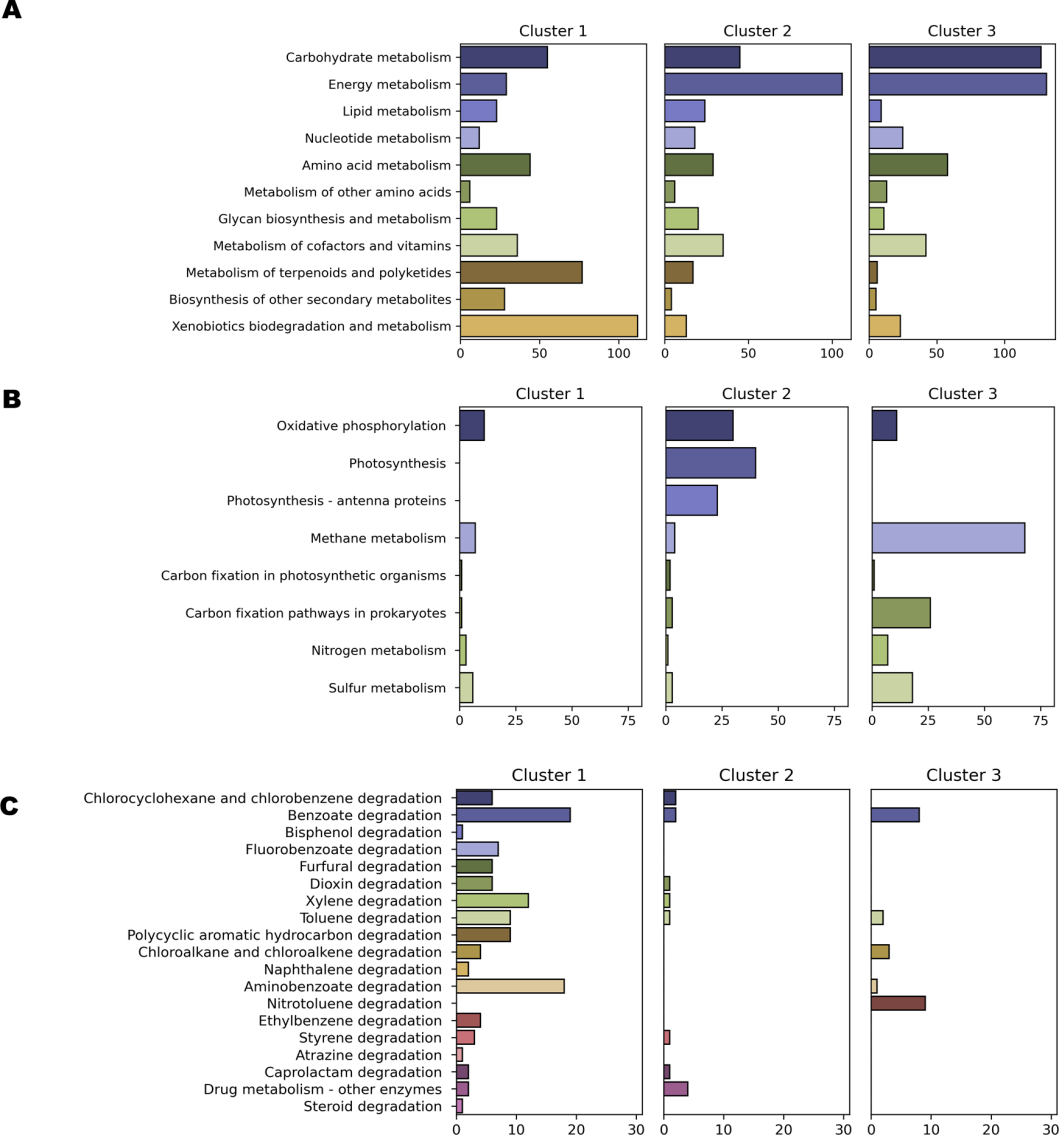
KEGG pathways associated with cluster-specific marker genes. Number of cluster-specific marker genes belonging to the different KEGG metabolism pathways (**A**), Energy metabolism pathways (**B**), and xenobiotics degradation and metabolism pathways (**C**).

Marker KOs were identified for each cluster to uncover the differentially abundant functional traits driving cluster formation. Marker KOs were assigned if they had a median log_2_(fold-change) greater than 2 and a Bonferroni corrected *P*-value < 0.001, both metrics were robust to false-positives when compared to random cluster assignment (Fig. S6). A total of 2,546 marker KOs were identified with 684, 945, and 917 belonging to Clusters 1, 2, and 3. Clusters 2 and 3 were enriched in energy metabolism-related KOs (106 in Cluster 2 and 131 in Cluster 3 vs 29 in Cluster 1), while Cluster 1 had more marker KOs related to the metabolism of terpenoids and polyketides (77 in Cluster 1 vs 17 in Cluster 2 and 6 in Cluster 3) as well as xenobiotics degradation and metabolism (112 in Cluster 1 vs 13 in Cluster 2 and 23 in Cluster 3) (Fig. 2A). Specifically, Cluster 1 had 76 markers related to polyketide biosynthesis and 34 related to antimicrobial resistance (Fig. S5). Within the energy metabolism category, Cluster 2 was dominated by photosynthesis-related marker KOs (*n* = 40), while Cluster 3 was dominated by marker KOs related to methane metabolism (*n* = 68), specifically methanogenesis (Fig. 2B). Cluster 3 also contained 26 marker KOs associated with carbon fixation pathways in prokaryotes and 18 with sulfur reduction metabolism. Within the xenobiotics degradation and metabolism category, Cluster 1 had 19 marker KOs associated with benzoate degradation, 18 with aminobenzoate degradation, and 12 with xylene degradation (Fig. 2C). Many of these KOs were annotated as dioxygenases associated with a variety of aromatic compounds that could be derived from lignin.

Beyond KOs related to metabolism, we found cluster-specific marker KOs associated with other core cellular processes such as transcription and translation. For example, Clusters 2 and 3 had marker KOs in the categories of ribosome, ribosome biogenesis, and transcription machinery (Fig. S7). Cluster 2 also had 90 marker genes in the category of chromosome and associated proteins and Cluster 3 had 48 markers associated with the prokaryotic defense system which are mostly CRISPR-associated proteins (Fig. S7). Closer inspection of marker KOs in these categories revealed many specific to eukaryotic cell functioning in the case of Cluster 2 and archaeal cell functioning in Cluster 3. To validate this finding, we analyzed the taxonomic composition of cluster metagenomes. Cluster 2 metagenomes had a significantly higher proportion of contigs assigned to eukaryotes (Kruskal-Wallis: *P* < 1e−306; Dunn’s multiple comparisons: *P* < 1e−216) while Cluster 3 had significantly more assigned to archaea (Kruskal-Wallis: *P* < 1e−287; Dunn’s multiple comparisons: *P* < 1e−217) and Cluster 1 had more assigned to bacteria (Kruskal-Wallis: *P* < 1e−308; Dunn’s multiple comparisons: *P* < 1e−251) (Fig. S5). Interestingly, Cluster 2 was also enriched in viral contigs (Kruskal-Wallis: *P* < 1e−308; Dunn’s multiple comparisons: *P* < 1e−105) (Fig. S5D). Despite these differences, all clusters were still dominated by bacterial contigs with a median prevalence of 99.0% in Cluster 1, 95.2% in Cluster 2, and 95.4% in Cluster 3 (Fig. S5A).

**Fig 3:**
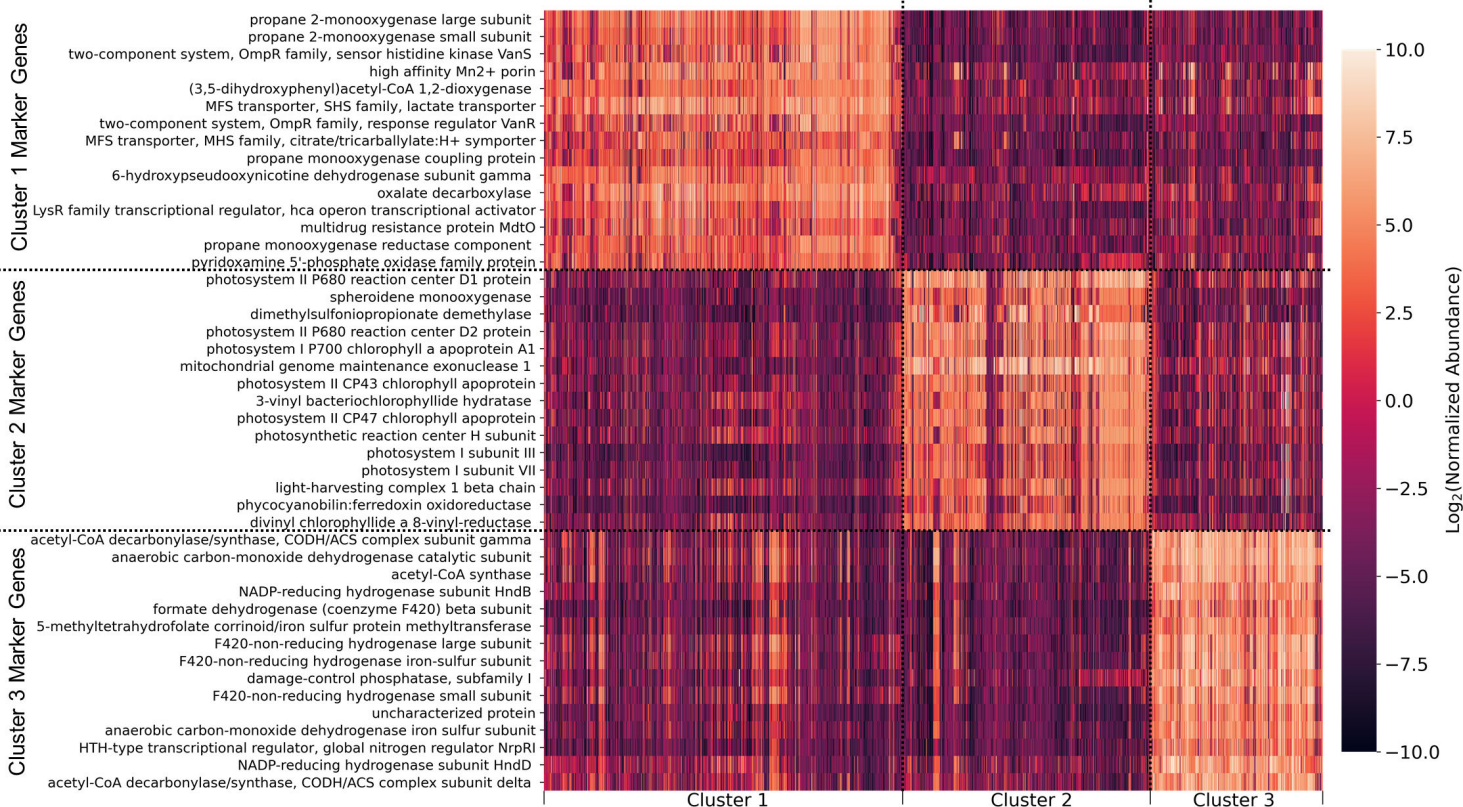
Top 15 cluster marker genes based on median fold-change. Each row represents a gene, and each column represents a metagenome, sorted by cluster. Cells are colored according to the clr normalized KO abundance in each sample, where zero represents a KO abundance equal to the geometric mean of the sample.

To look at specific examples of marker KOs, we selected the top 15 based on median fold-change for further investigation. The heatmap shows the abundance of top genes (rows) in every sample (columns) and reveals a clear separation of clusters and highlights the uniqueness of these KOs (Fig. 3). Surprisingly, four of the top Cluster 1 marker KOs were annotated as the propane monooxygenase (*prm*), with other KOs associated with antibiotic resistance and synthesis, transport of manganese, lactate, and citrate. Cluster 2 markers were dominated by genes related to photosynthesis and the biosynthesis of related pigments. One KO was also related to the metabolism of dimethylsulfoniopropionate, an important algal metabolite that sustains many marine bacteria. Cluster 3 top KOs were related to anaerobic energy metabolisms; for example, the carbon monoxide dehydrogenase/acetyl-CoA synthase (CODH/ACS) complex, several hydrogenases and more intriguingly, a damage-control phosphatase, and a transcription factor related to nitrogen regulation.

### Functional clustering separates metagenomes by underlying GC content

**Fig 4:**
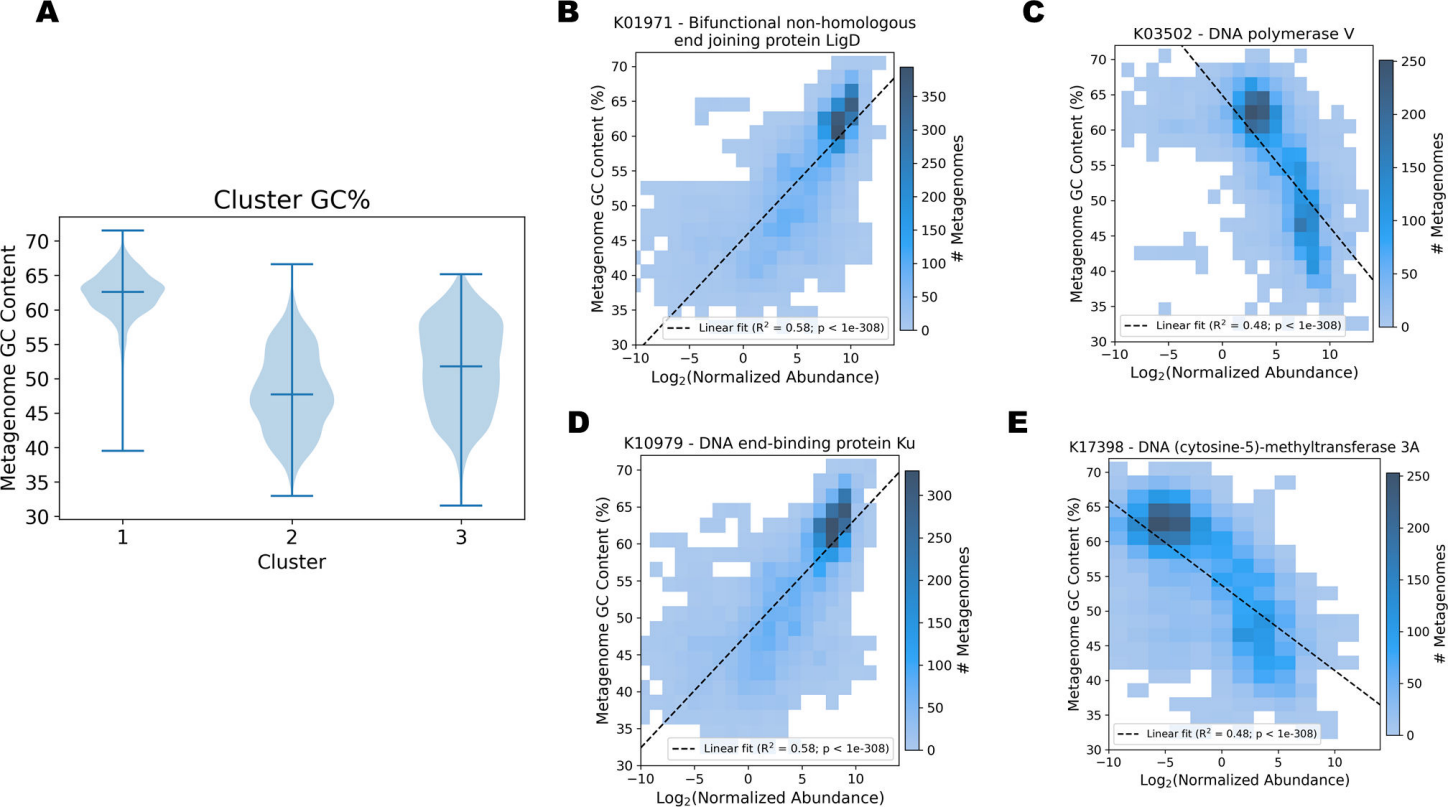
GC content variation and correlated genes of interest. (**A**) Distribution of assembled metagenome GC content in different clusters. Binned scatter plots showing GC content vs the abundance of the bifunctional non-homologous end-joining protein LigD (**B**), DNA polymerase V (**C**), DNA end-binding protein Ku (**D**), and DNA (cytosine-5)-methyltransferase 3A (**E**).

Guanine-cytosine (GC) content is a fundamental characteristic of DNA sequences that represents the proportion of GC vs AT base pairings. Exploration of data set metadata revealed that the metagenomes from the functional clusters had significantly different GC contents (Kruskal-Wallis: *P* < 1e−308; Dunn’s multiple comparisons: *P* < 1e−25) despite being formed solely based on KO abundance and no other sequence characteristics (Fig. 4A). Cluster 1 metagenomes had consistently high GC contents, while Cluster 2 and Cluster 3 GC contents were lower and more varied. To gain insight into the potential functional drivers of this trait, we performed simple linear regression to identify highly correlated genes. Two genes related to non-homologous end-joining DNA repair, *LigD* and *Ku*, were positively correlated with GC content (Fig. 4B and D). In contrast, DNA polymerase V (*polV*), which is associated with the SOS response to UV damaged DNA (27), and a DNA cytosine methyltransferase KO were negatively correlated with GC content (Fig. 4C and E). Aside from genes related to DNA processes, four genes related to trehalose metabolism were positively correlated with GC content and a pseudaminic acid synthase and Na^+^-transporting NADH:ubiquinone oxidoreductase complex were among the most negatively correlated.

### Sub-clustering reveals finer scale differences within the functional landscape

To investigate how our approach performed on a more similar set of metagenomes, we further divided each of the three main clusters into two sub-clusters. The sub-clusters appeared as cohesive groups within the UMAP of the data set (Fig. S11B, D and F). Cluster 2 was clearly separated into marine and freshwater dominated sub-clusters, Cluster 3 produced one sub-cluster enriched in digestive systems and engineered environment samples, while Cluster 1 was split relatively evenly with respect to ecosystem label (Fig. S11A, C and E). Top marker KOs, based on median fold-change, for Cluster 2 were associated with the metabolism of compatible solutes ectoine and betaine in the marine sub-cluster, while KOs associated with magnesium, sulfate, potassium transport were enriched in the freshwater sub-cluster. The Cluster 3 digestive system associated sub-cluster had numerous marker KOs associated with sporulation. Finally, Cluster 1 markers were associated with either fungal metabolism (e.g., chitin and 1,3-beta-glucan synthesis, yeast amino acid transporter, and other eukaryotic cell functions) in one sub-cluster while a multicomponent K^+^:H^+^ antiporter involved in bacterial pH adaptation and the Rnf H^+^/Na^+^-translocating ferredoxin:NAD^+^ oxidoreductase complex were top markers in the other sub-cluster.

## DISCUSSION

Metagenomics is an increasingly valuable tool for interrogating the structure and function of microbiomes, nonetheless analysis of this complex data across large sample sets remains challenging. Here, we focus on KO abundance profiles as a consistent and low-dimensional embedding of functional potential to enable comparison across a large set of metagenomes. This dimension reduction results in our entire data set occupying a few hundred megabytes of storage, while the associated raw sequencing data likely occupies hundreds of terabytes. Some of the earliest metagenomics studies made similar observations of ecosystem-specific clustering of function using only a few samples (13, 14). We extend these findings to a much larger and more diverse data set and analyze cluster-specific marker KOs. Splitting the markers into their associated KEGG pathway categories highlighted which metabolic functions defined each cluster, and by extension their respective environments. Broadly, we found that the availability of oxygen for respiration and light for photosynthesis were strong drivers of differentiation (aquatic vs terrestrial vs sludge). Furthermore, KOs related to domain-specific cellular functioning were enriched in different clusters (i.e., archaea in Cluster 3, eukaryotes in Cluster 2). Finally, the top cluster-specific marker KOs pointed at several interesting adaptations to different environments.

Cluster 1 was dominated by soils and characterized by marker genes related to the degradation of a diverse set of aromatic compounds as well as antibiotic synthesis and resistance. There were marker genes associated with the breakdown of vanillate, syringate, catechol, and anthranilate which are all key intermediates in the bacterial degradation of lignin (28). Lignin is an important component of plant cell walls that accounts for approximately 30% of global organic carbon stocks (29), as such it represents a key carbon source in soils (28, 30). Interestingly, the *prm* propane monooxygenase was one of the most differentially abundant genes in Cluster 1. In addition to propane oxidation (31), studies have associated this enzyme with *N*-nitrosodimethylamine degradation (32) and 1,4-dioxane degradation (33), suggesting that it may participate in the breakdown of other recalcitrant compounds in the environment. The apparent importance of this enzyme complex in soils warrants a more detailed exploration of its diversity and function. Previous reports have identified soils as rich reservoirs of both antibiotics (13, 34) and antibiotic resistance (35, 36), and our results reinforce that this as a defining functional characteristic of soils compared to other ecosystems. Lastly, sub-clustering suggested soils could be grouped based on fungal vs bacterial abundance, possibly driven by pH, which has been shown to be one of the strongest drivers of microbial community assembly in soils (15, 37).

Cluster 2 contained almost exclusively freshwater and marine samples and was clearly distinguished by genes related to photosynthesis and associated pigments. This cluster had the highest proportion of eukaryotic contigs and had marker KOs associated with eukaryotic cell biology presumably from an abundance of algae. Aside from photosynthesis, dimethylsulfoniopropionate (DMSP) demethylase was one of the top marker genes. DMSP is an important osmolyte for phytoplankton that can account for as much as 10% of their fixed carbon and also acts as a key carbon source for bacteria (38). The identification of the DMSP demethylase as one of the top marker genes illustrates the extent to which phototrophs shape the food web in aquatic systems. Sub-clustering divided freshwater and marine metagenomes based on the metabolism of compatible solutes and transport of inorganic ions, highlighting the unique adaptations of microbial life to aquatic environments of differing ionic strength. Furthermore, these observations illustrate how the clustering method can reveal finer scale differences in functional potential when the broader environmental context is held constant.

Cluster 3 consisted of a variety of anaerobic environments from aquatic sediments to anaerobic digestors and gut microbiomes. This points to the conservation of anaerobic metabolisms (i.e., methanogenesis, sulfur reduction, and fermentation) in spite of the broader environmental context. Indeed, an evolutionary study of the CODH/ACS complex, a key enzyme in the Wood-Ljungdahl pathway of carbon fixation and the top marker KO for Cluster 3, revealed an astounding level of conservation across many anaerobic bacterial and archaeal lineages (39). Cluster 3 harbored numerous marker genes specific to archaeal cell functioning and had the highest proportion of archaeal contigs, highlighting the unique dominance and adaptation of this domain of life in anaerobic settings. We also found many CRISPR-associated genes as markers for Cluster 3, which is in alignment with a study of isolate genomes that found CRISPRs were more prevalent in anaerobes compared to aerobes (40). This suggests that defense against phage predation is especially important in anaerobic settings in contrast to Cluster 1 where genes for antibiotic production and resistance were uniquely abundant. Cluster 3 had one sub-cluster associated with digestive system samples and spore formation. These environments may experience fluctuating conditions that require sporulation for the survival of anaerobes compared to the anaerobic sediments, found in the other sub-cluster, that have more steady environmental conditions.

We observed significant differences in GC content across functional clusters, which corroborates previous reports of metagenome GC content partitioning across environments (41, 42). This phenomenon has been discussed from a variety of viewpoints, such as differences in carbon to nitrogen ratios, oxygen requirements, genome size, and DNA repair pathway (43). Using our functional gene approach, we found that NHEJ-related genes were correlated with high GC content while *polV* and a cytosine methyltransferase were correlated with low GC content. The role that NHEJ and *polV* play in shaping GC content has been explored in isolate genomes (43, 44). In addition, methylated cytosine bases are mutational hotspots (45) that have been suggested to play a role in the evolution of GC content in vertebrate genomes (46). Our results suggest that differences in DNA repair and methylation play a role in shaping GC content even at the whole community level. This reinforces the notion that environmental conditions shape community functional potential, which, in turn, may contribute to the evolution of distinct genomic traits, such as GC content.

While this approach provides an insightful glimpse into the functional potential of microbial communities across ecosystems, it is still limited by our ability to comprehensively assemble and annotate short metagenomic sequencing reads. The sequence assembly difficulties are particularly acute in soil samples due to their immense diversity. Furthermore, we are only able to assign KOs to roughly 40% of genes in this data set. A key question becomes, is the composition of microbial dark matter (47), what we cannot assemble or annotate, vastly different from what we can assemble and annotate? Given the functional redundancy of microbial communities, it is possible that the elucidation of the “dark matter” will not significantly change our overall view of community function. Lastly, it is worth noting that metagenomes only tell us about functional potential and should not be misconstrued as proxies of actual activity, for which proteomics, transcriptomics, or stable-isotope probing would be more appropriate.

Overall, this study demonstrates the value of ortholog abundance profiles for representing microbial communities and supports their use in modeling community function. Ortholog abundance-based models have shown promise, for example, in explaining geochemical patterns of nutrient cycling in the ocean (17, 48, 49) and in predicting metabolic preference for sugars vs amino and organic acids in a collection of isolates (50). This approach takes advantage of the functional redundancy of microbial communities, that is the fact that functional traits are more stable than taxonomy (51, 52). Focusing on function simplifies the comparison across microbial communities as evidenced by this study, which includes thousands of metagenomes. The data set provides a valuable resource for the exploration of the functional gene landscape across a range of ecosystems. It is amendable to expansion with additional samples and metadata describing key parameters like pH, temperature, and oxygen availability. Integrating ortholog abundance profiles with quality metadata will bring us closer to a predictive understanding of how microbial communities assemble and function in the face of varying environmental conditions.

## Data Availability

Compiled ortholog abundance profiles and metadata with associated ascension numbers are available from the associated figshare project (https://figshare.com/projects/Metagenome_functional_clustering/187989). Analysis code can be found in the associated github repository (https://github.com/zflink/Metagenome_functional_clustering). Raw metagenomic data and ortholog abundance profiles are available from the JGI IMG/M database.

## SUPPLEMENTAL MATERIAL

Supplemental figures msystems.00573-24-s0001.docx Figures S1-S11.

ASM does not own the copyrights to Supplemental Material that may be linked to, or accessed through, an article. The authors have granted ASM a non-exclusive, world-wide license to publish the Supplemental Material files. Please contact the corresponding author directly for reuse.
