# Supplementary material for: Metagenomic clustering links specific metabolic functions to globally relevant ecosystems
Source: mSystems. 2024 Jul 9;9(8):e00573-24. doi: 10.1128/msystems.00573-24 (PMC11334424; doi:10.1128/msystems.00573-24)
Supplement: Supplemental figures — Figures S1-S11. [file msystems.00573-24-s0001.docx]

SUPPLEMENTARY INFORMATION

TITLE: Metagenomic clustering links specific metabolic functions to globally relevant ecosystems

SHORT TITLE: Metagenomic clustering across ecosystems

**Zachary Flinkstrom^1^, Samuel Bryson^2^, Pieter Candry^1,3^, Mari-Karoliina H. Winkler^1^**

^1^ Department of Civil and Environmental Engineering, University of Washington, Seattle, WA

^2^ Phase Genomics, Seattle, WA

^3^Current address: Laboratory of Systems and Synthetic Biology, Wageningen University & Research. 6708 WE, Wageningen, The Netherlands

*Correspondence to: Zachary Flinkstrom, Civil and Environmental Engineering, University of Washington, 201 More Hall, Box 352700, Seattle, WA 98195-2700, USA; phone: +1-207-251-0908; E-mail: zflinky@gmail.com

**Supplementary figures**

**
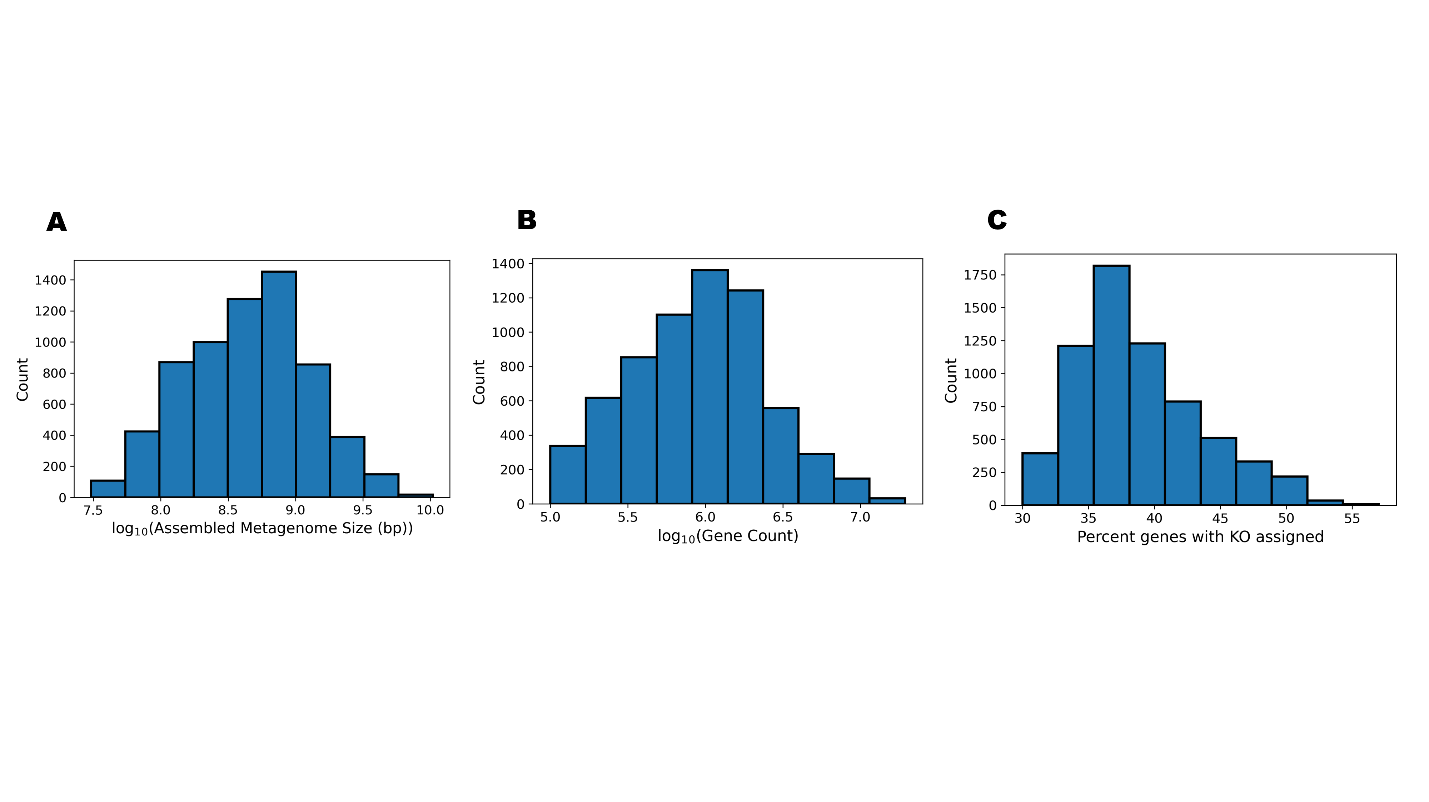
**

**Figure S1 – Characteristics of dataset metagenomes.** (A) Histogram of assembled metagenome size in base pairs. (B) Histogram of assembled metagenome gene count. (C) Histogram of percent of genes with KO assigned.

**
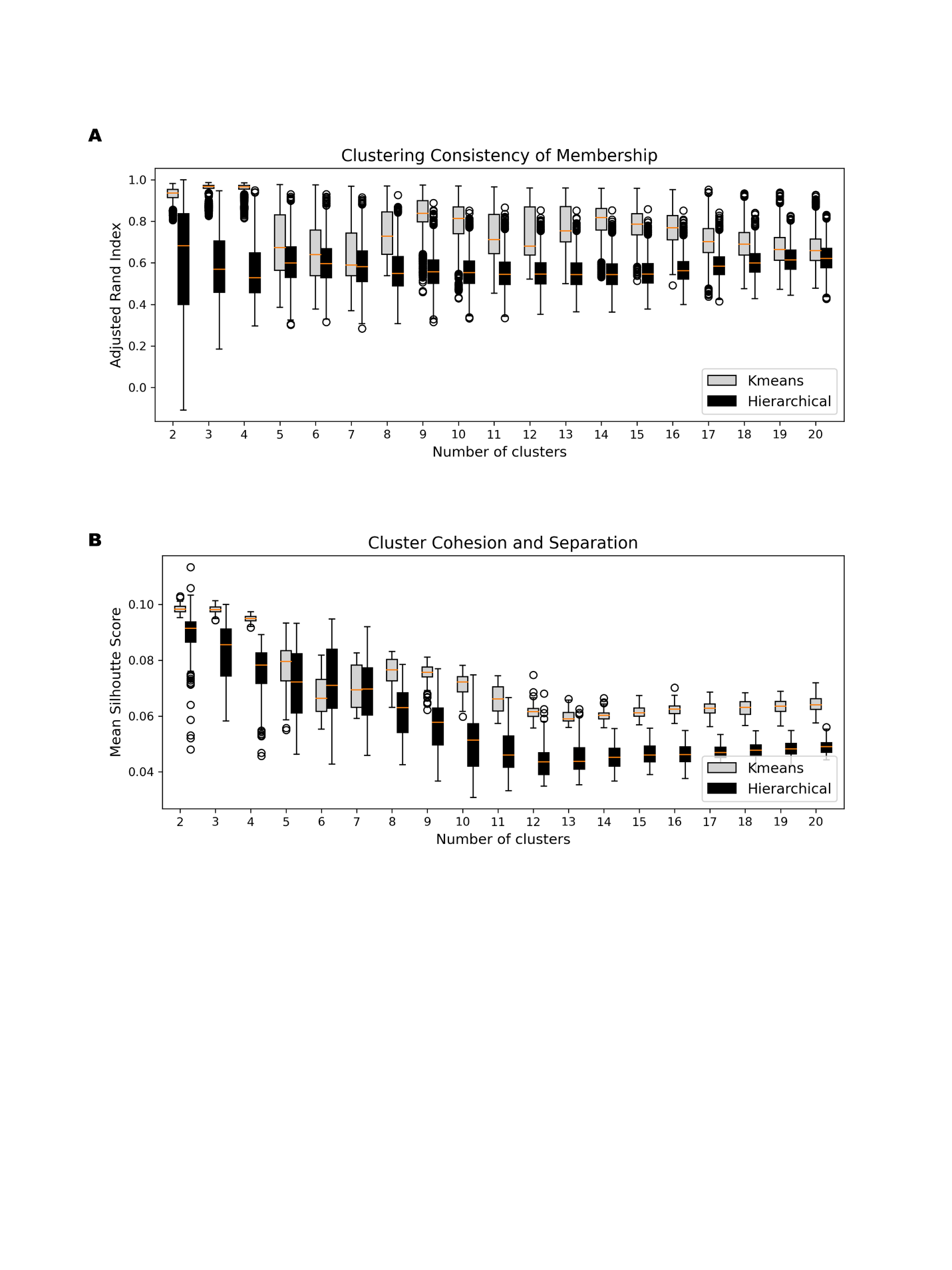
**

**Figure S2 – Bootstrap testing of k-means and Ward’s hierarchical clustering methods over a range of cluster numbers.** (A) Adjusted Rand Index results from clustering of 100 bootstrap samples of the dataset over a range of 2 to 20 clusters. (B) Mean Silhouette Coefficient results from the same clusters.


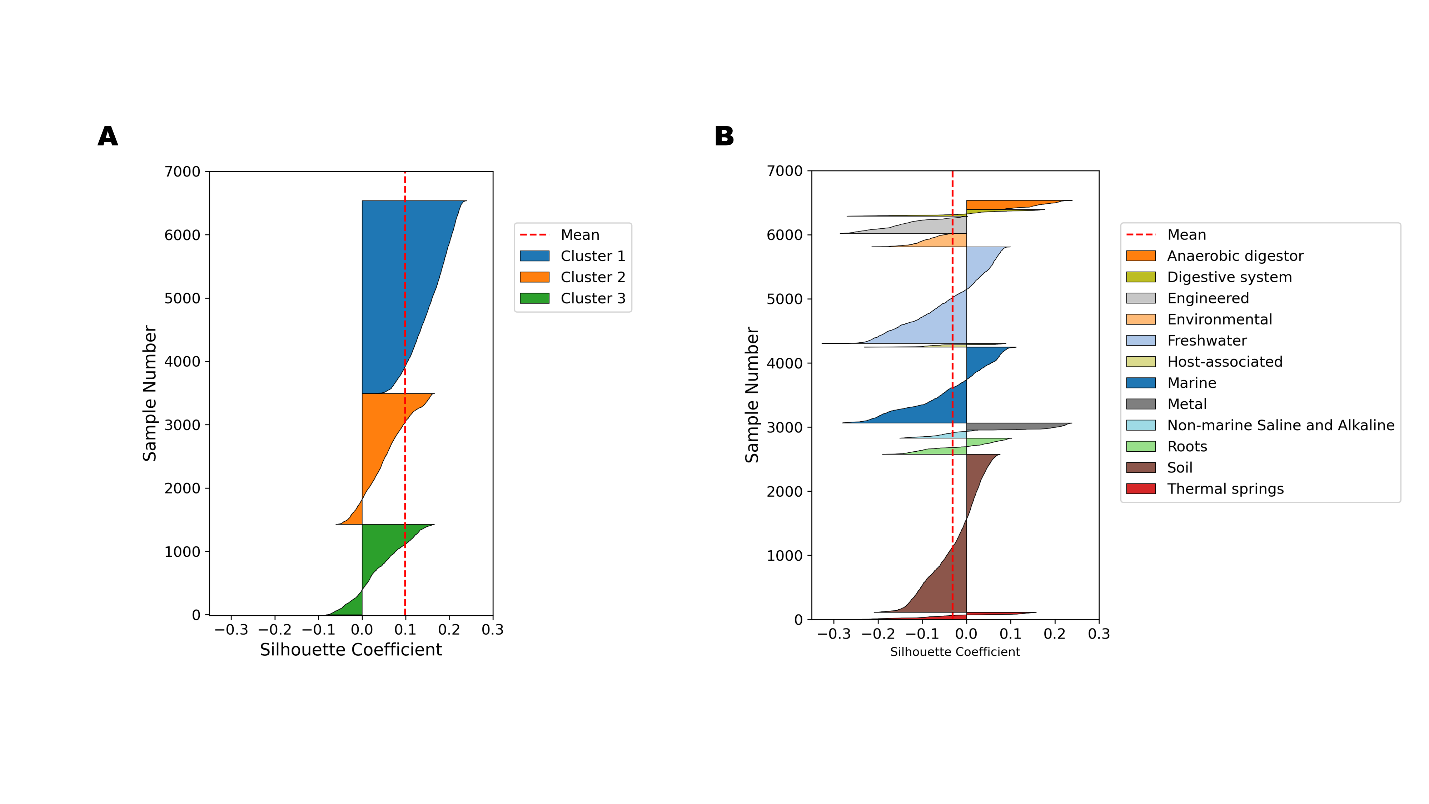


**Figure S3 – Distribution of Silhouette coefficients across the dataset.** Silhouette coefficient values for each sample based on k-means (A) versus ecosystem label grouping (B). Mean Silhouette Coefficient for all samples is shown with red dashed line.


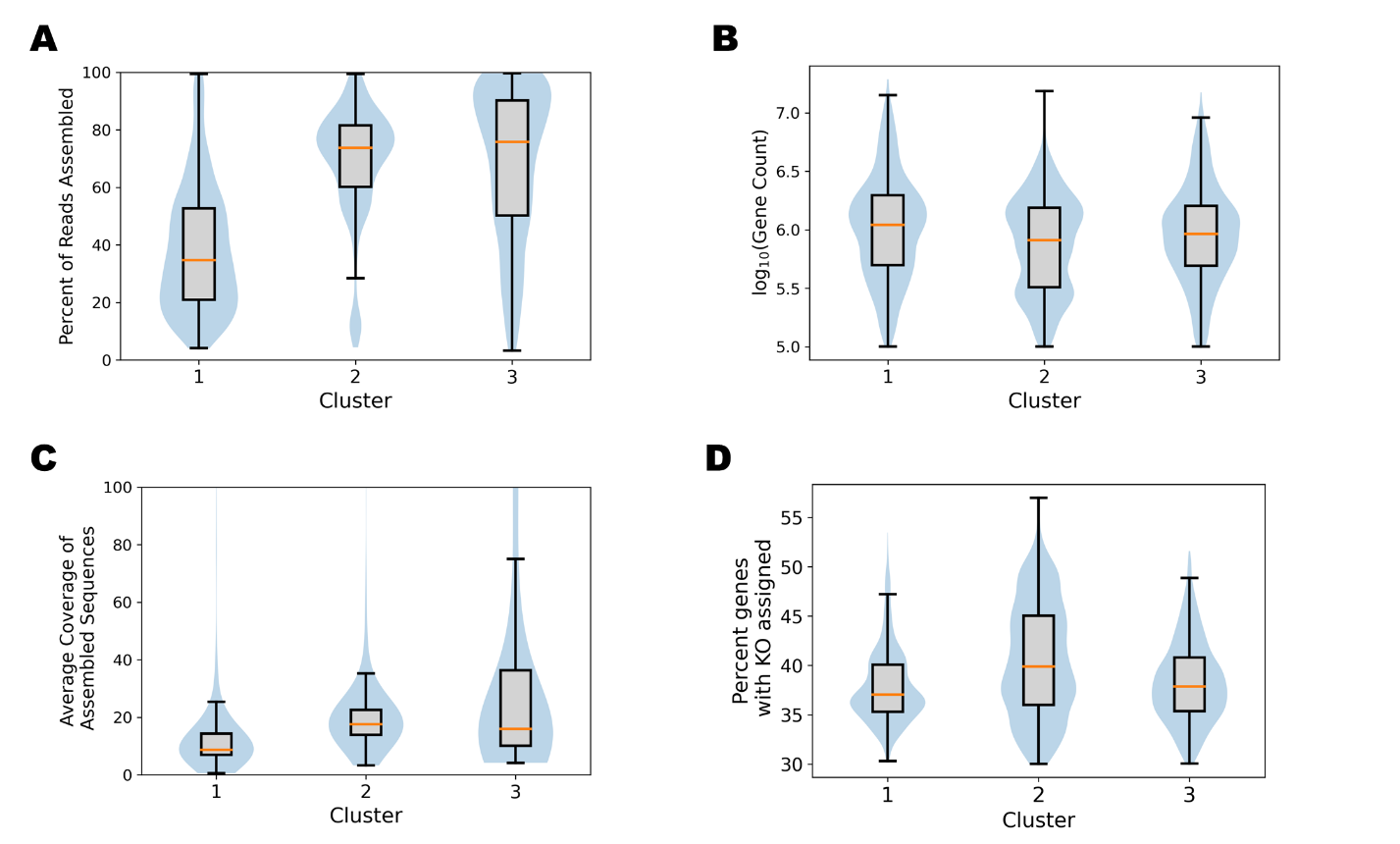


**Figure S4 – Characteristics of metagenomes separated by cluster assignment.** Boxplot and violin plots, broken down by cluster, showing (A) the percent of sequencing reads assembled, (B) log10 of gene count, (C) average coverage of assembled sequences, and (D) percent of genes with a KO assigned.


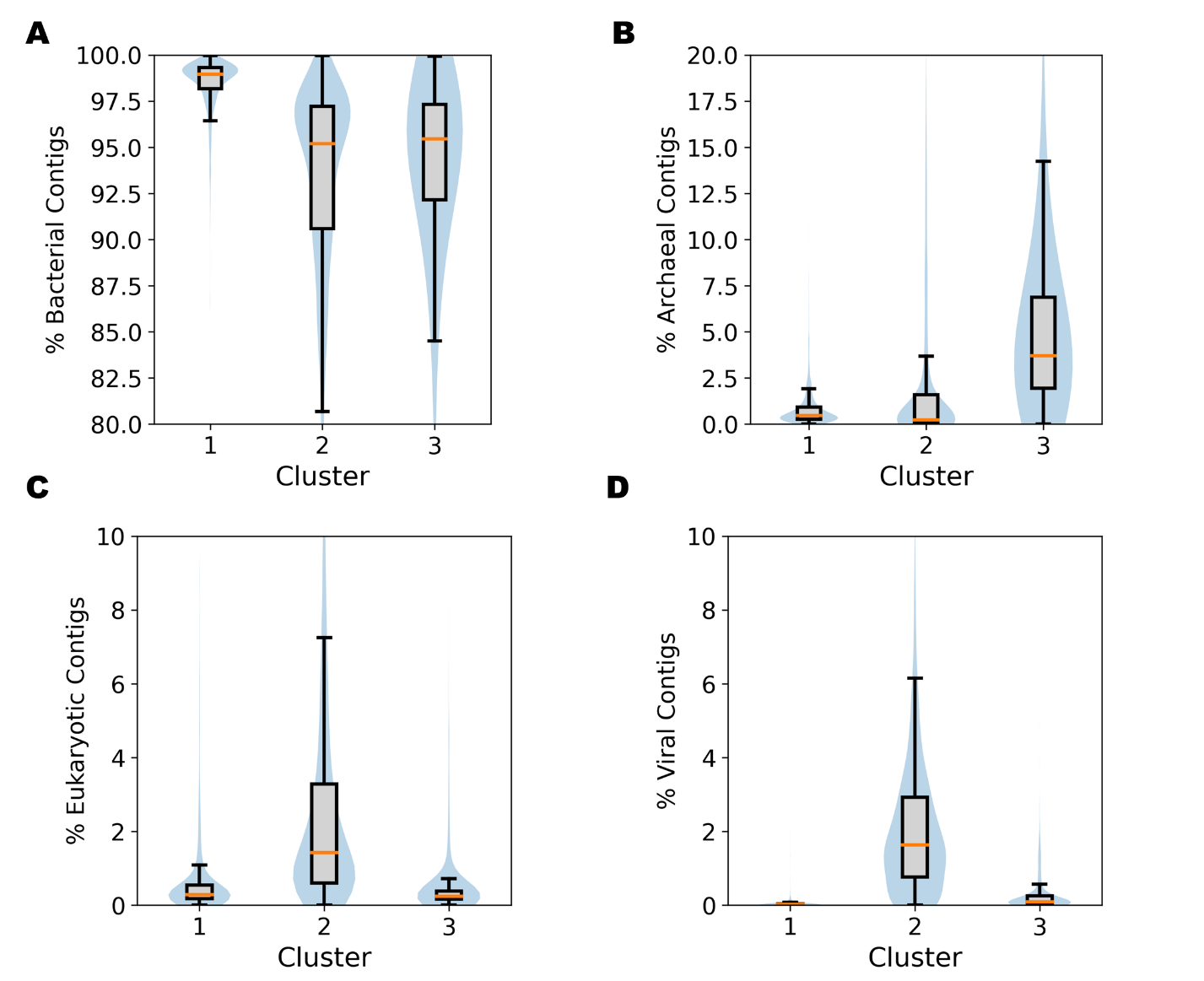


**Figure S5 – Domain-level taxonomic composition of metagenome contigs in each cluster.** Boxplot and violin plots showing (A) the percent of contigs assigned to bacteria, (B) archaea, (C) eukaryotes, and (D) viruses. Data derived from JGI IMG/M’s taxonomic composition tables which assign contig phylogeny based on database homology search of protein-coding genes.

**
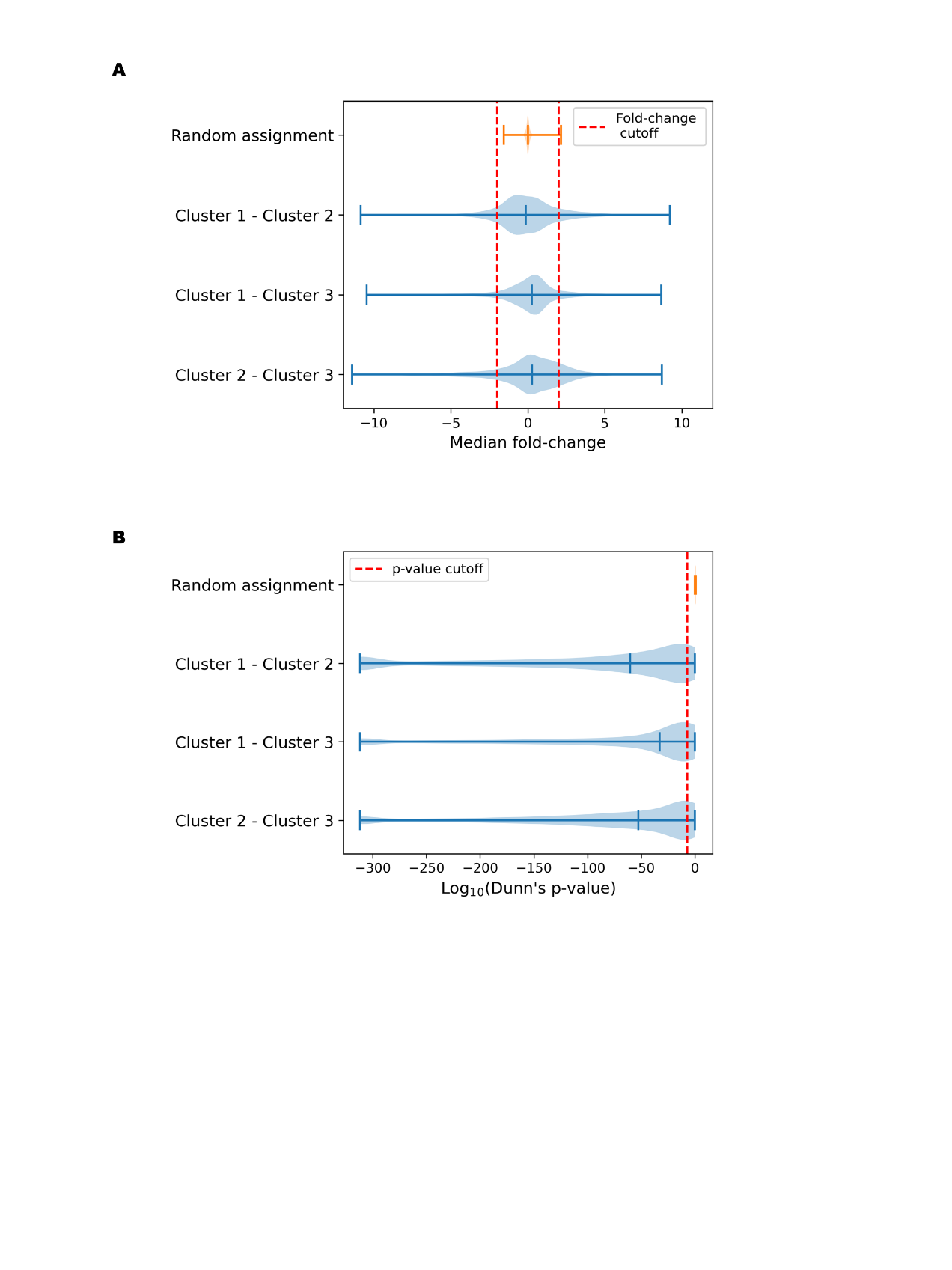
**

**Figure S6 – Assignment of cluster-specific marker genes.** (A) Distributions of median fold-change in gene abundance between clusters compared to if clusters were randomly assigned with fold-change cutoff shown in red. (B) Distributions of Dunn’s test p-values f compared to random assignment with p-value cutoff shown in red. Results based on 100 random cluster assignments.

**
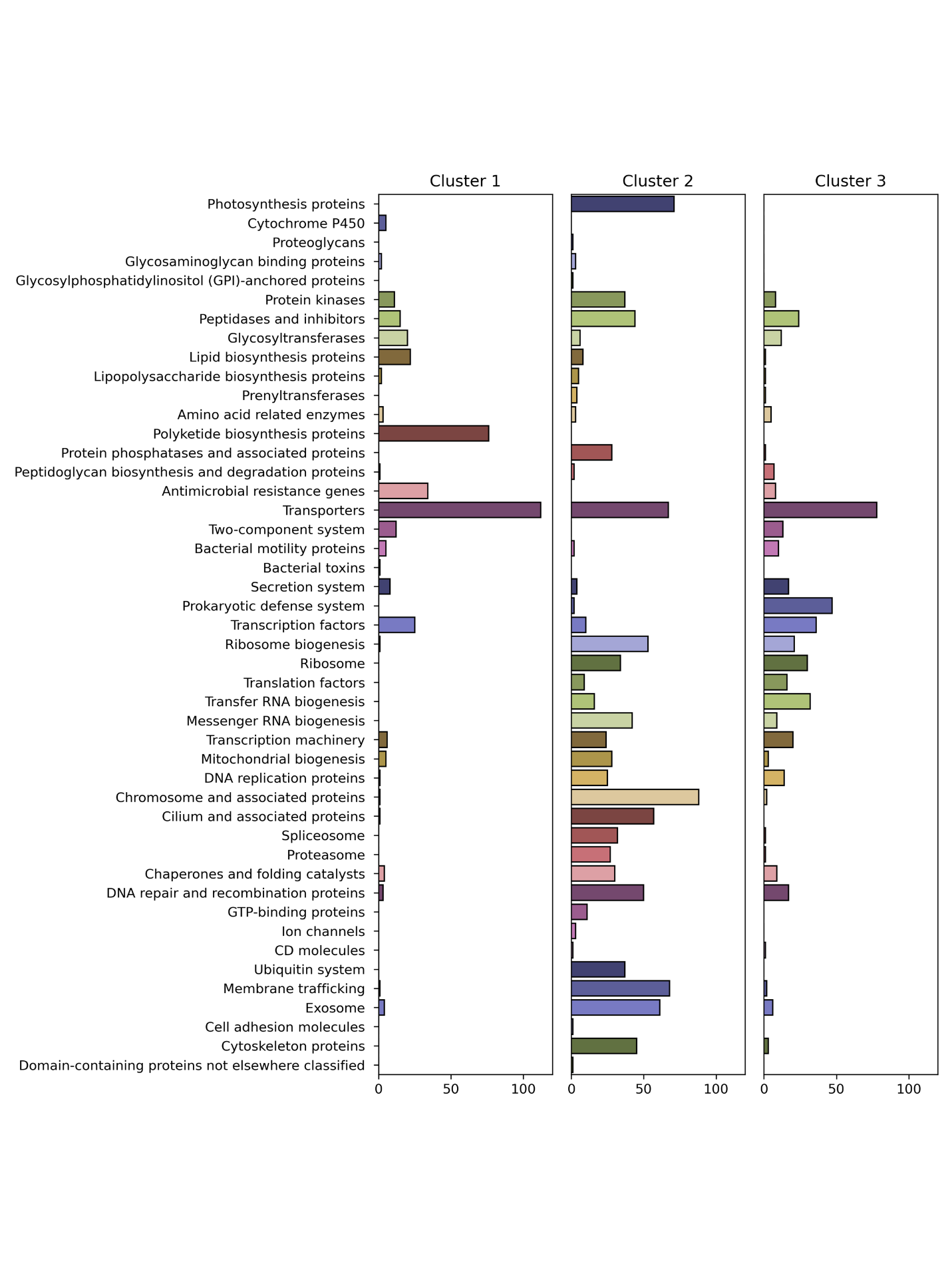
**

**Figure S7 – Number of cluster-specific marker genes associated with KEGG Brite Categories.**

**
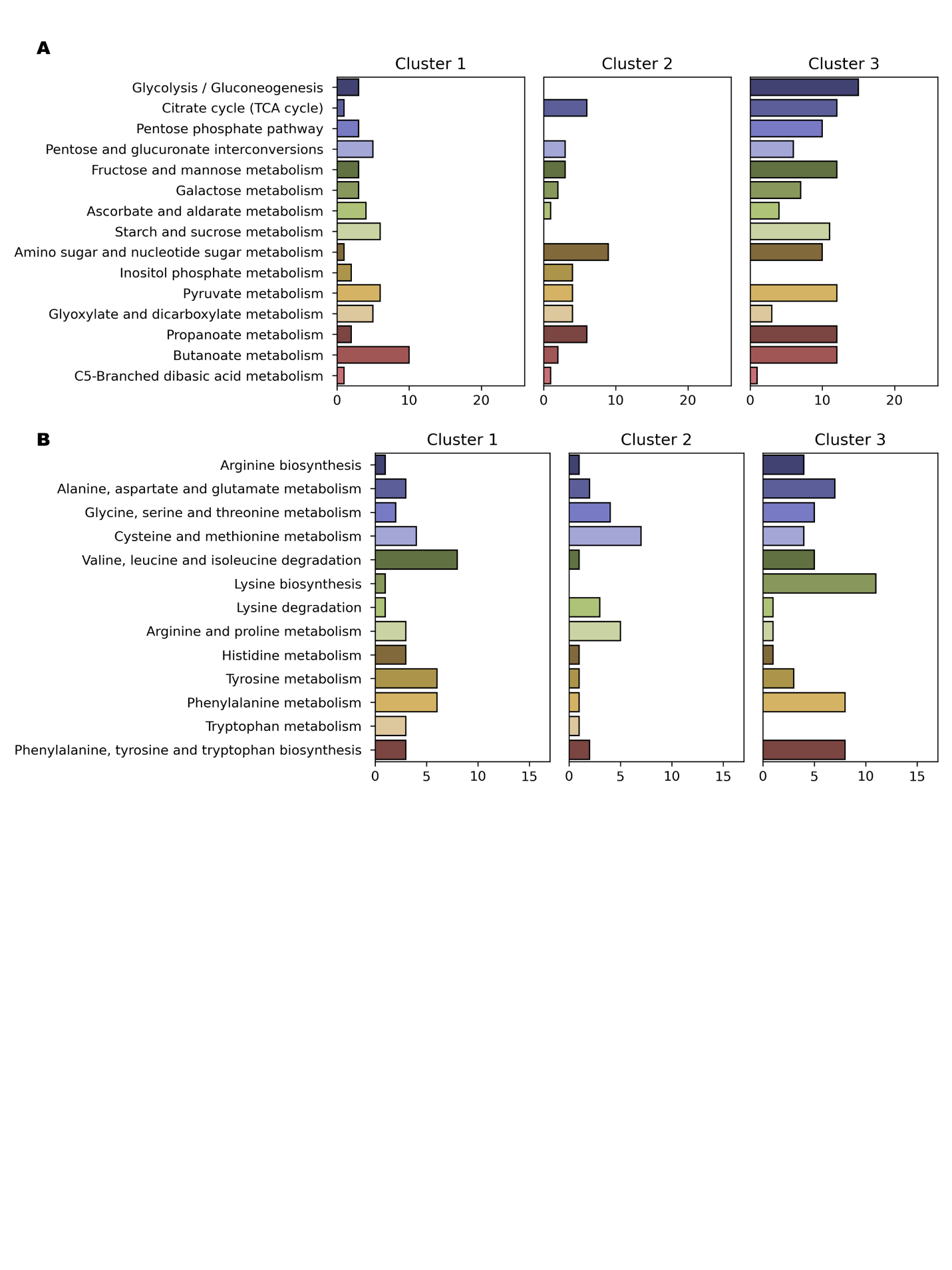
**

**Figure S8 – Number of cluster-specific marker genes associated with KEGG carbohydrate metabolism (A) and amino acid metabolism (B).**

**
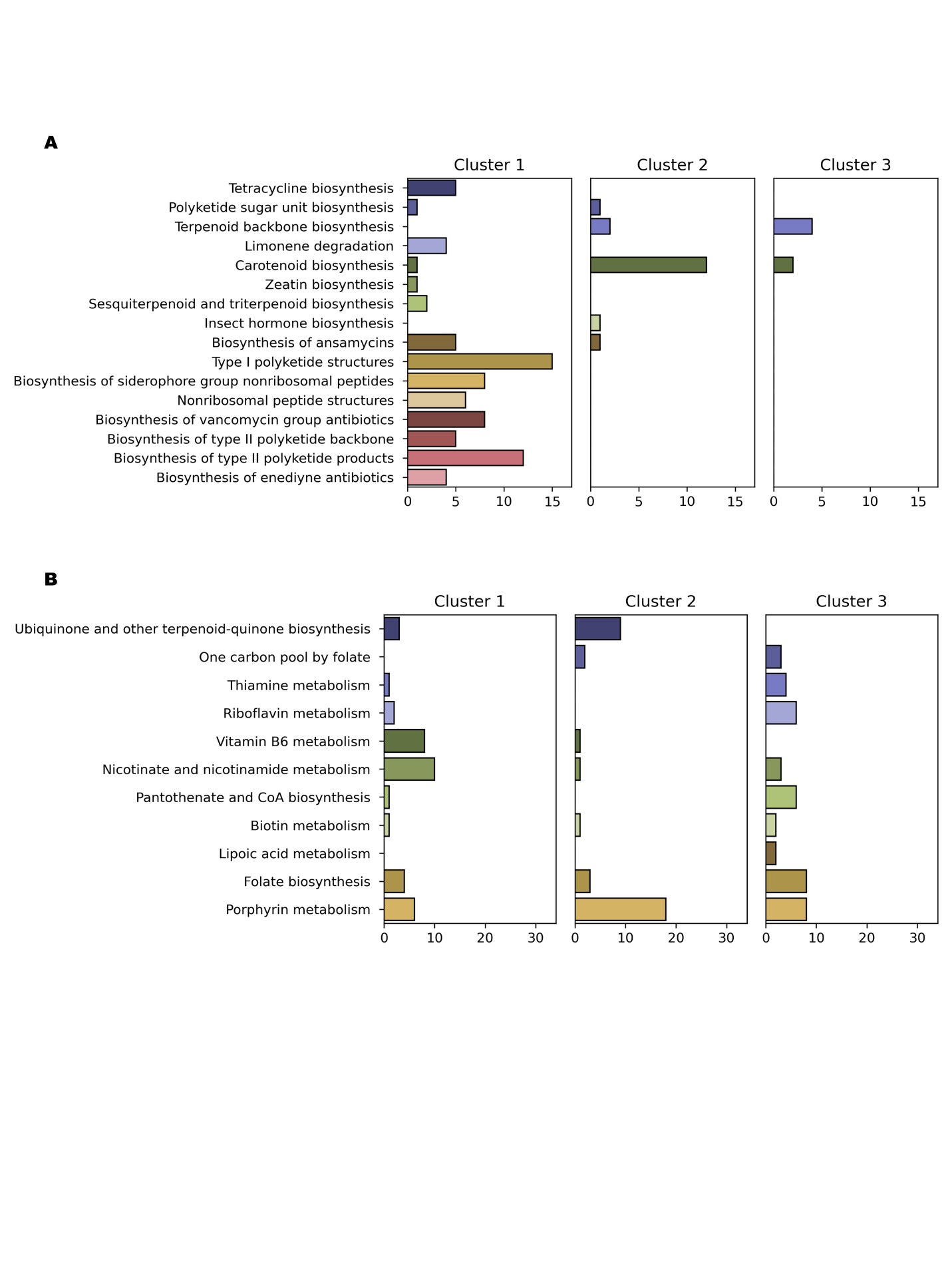
**

**Figure S9 – Number of cluster-specific marker genes associated with KEGG metabolism of terpenoids and polyketides (A) and vitamins and cofactors metabolism (B).**

**
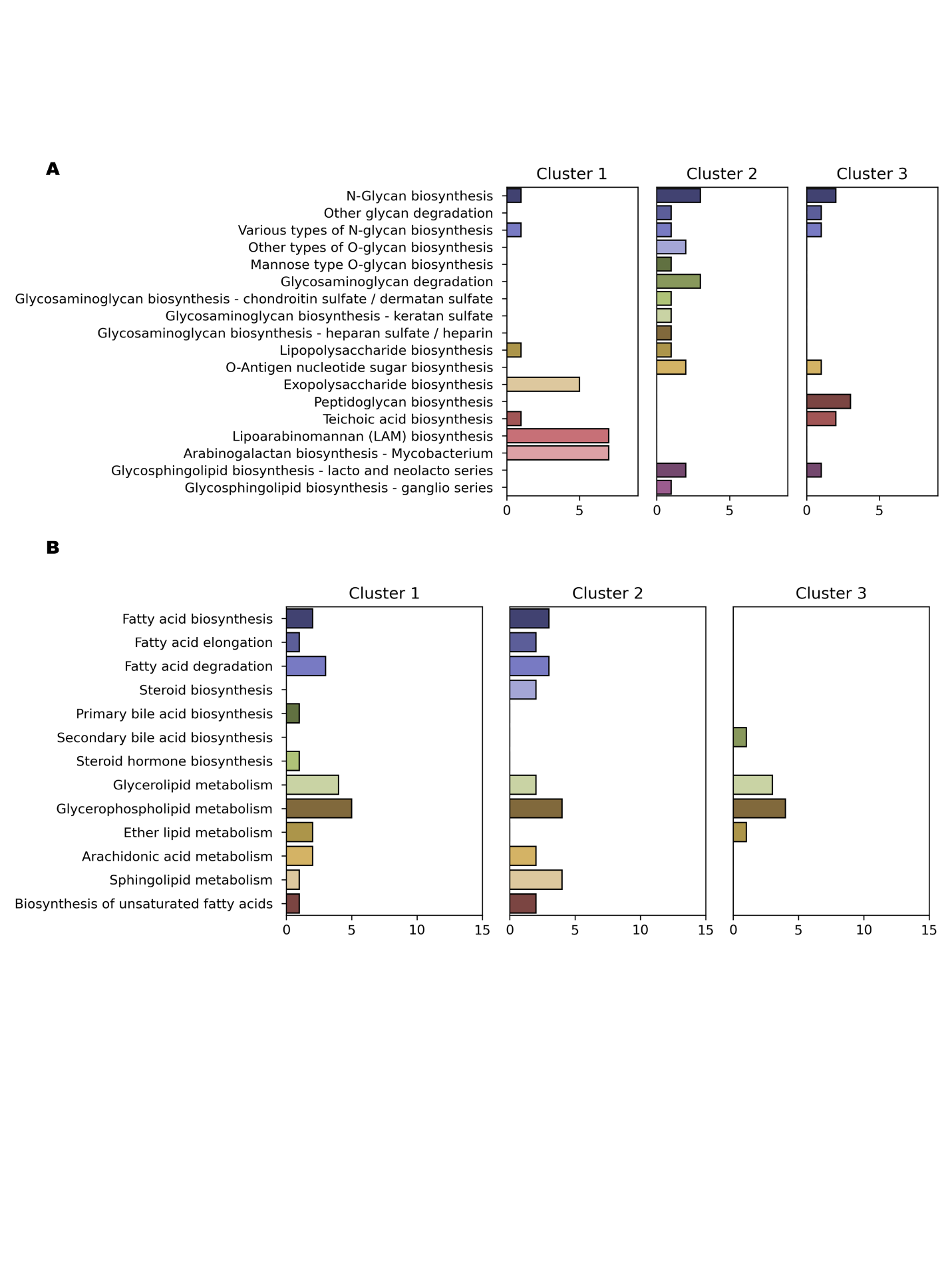
**

**Figure S10 – Number of cluster-specific marker genes associated with KEGG glycan biosynthesis and metabolism (A) and lipid metabolism (B).**

**
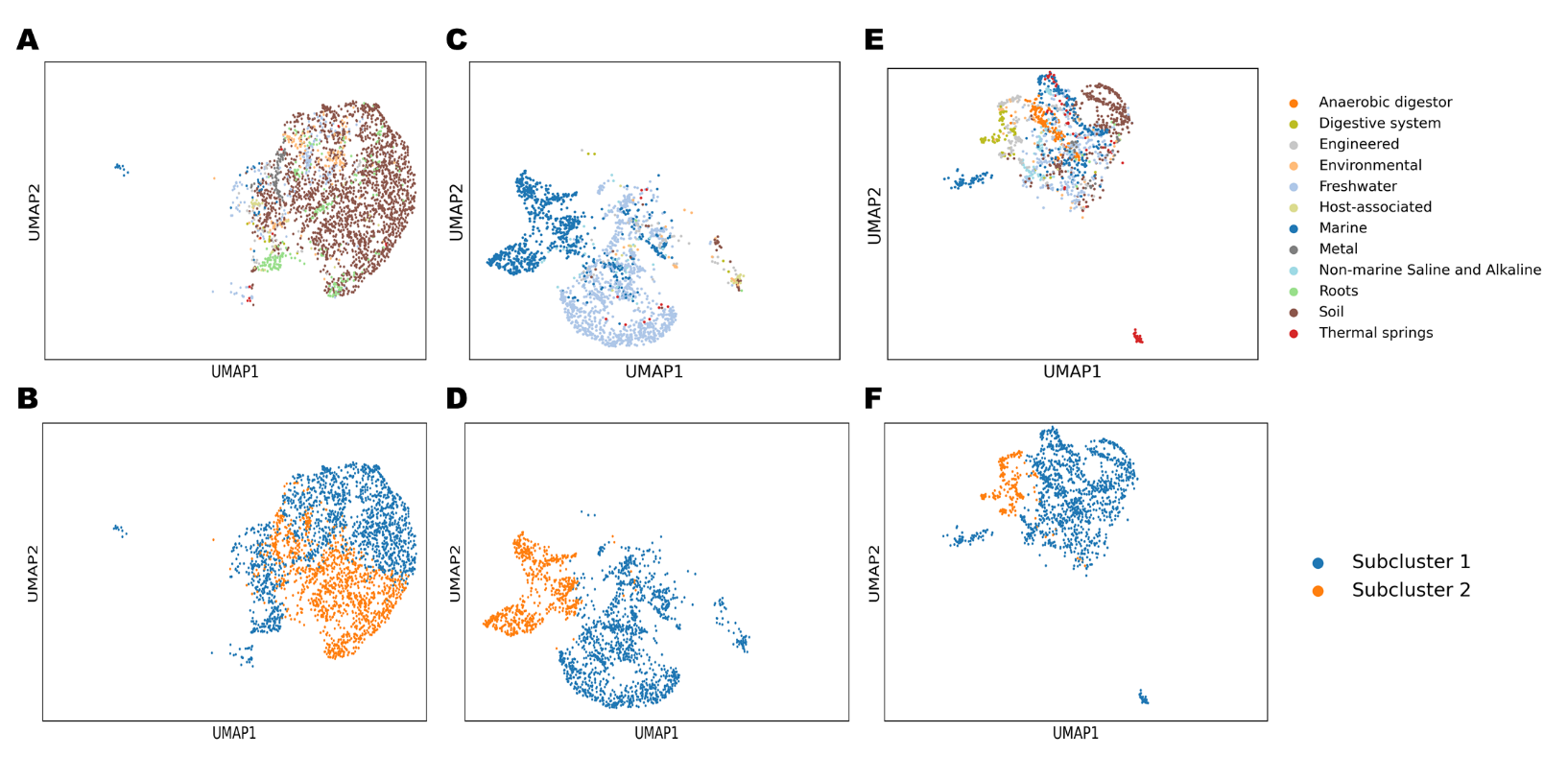
**

**Figure S11 – Sub-clustering analysis.** UMAP plots, using the same projection coordinates as Fig. 1B, showing just Cluster 1 (A) and the sub-clusters of Cluster 1 (B), Cluster 2 (C) and the sub-clusters of Cluster 2 (D), and Cluster 3 (E) and the sub-clusters of Cluster 3 (F).
